# The Hospital. Nursing Section

**Published:** 1902-07-19

**Authors:** 


### The Hospital.

### Nursing Section.

Contributions for this Section of "The Hospital" should be addressed to the Editor, "The Hospital" Nursing Section, 28 & 29 Southampton Street, Strand, London, W.C.

No. 825.—Vol. XXXII. *SATURDAY, JULY* 19, 1902.

### Notes on News from the Nursing World.

### NURSES ON THE ROYAL YACHT.

It has been a matter of comment that, though the King was sufficiently convalescent to undertake the journey by rail to Portsmouth, where he embarked on board the Royal Yacht on Tuesday, he had both of his nurses to accompany him. The fact has been construed by sensation-mongers into an admission that the health of his Majesty is not really so satisfactory as the public have been given to understand. This is not the case, and the actual nursing required by his Majesty is not of an arduous character. But, evidently, so long as the services of nurses are needed, they must be available at all hours. The presence of two nurses on the Royal Yacht is not, therefore, a matter to cause anxiety, and we can well believe that his Majesty is glad that the nurses who have borne the brunt of the work should now enjoy the benefit of the sea breezes and change of air. Only one nurse together with the Queen and the two surgeons accompanied the King in the ambulance, the other nurse having left Buckingham Palace earlier, taking with her the pillows and rugs. A slight mishap occurred to this carriage on its reaching Victoria Station; the horses stumbled and both came down, but happily no one was hurt.

### NURSES FROM SOUTH AFRICA.

Among the nurses on board the *Assaye*, which arrived at Southampton last week, were Supt. L. M. Stewart, of the Army Nursing Service, Sisters B. Shepley, A. Ridley, V. M. Carter, of the Army Nursing Service Reserve; and Sister F. E. Bickmore, civil. Miss Stewart, who requires three months' leave, and Miss Shepley and Miss Ridley, who require thirty days, return to South Africa. Miss Carter requires two months' leave, and her return is doubtful; while Miss Bickmore does not wish to go back at all. On board the *Walmer Castle*, which also arrived at Southampton last week, were Sister L. E. C. Steen, of the Army Nursing Service, who requires three months' leave, and Sister L. E. March, locally engaged for the voyage, who wishes to return to South Africa after one month's leave.

### HOURS IN THE TROPICS.

An important point in connection with nursing in the Tropics was raised by Mr. Hugh Clifford at the annual meeting of the Colonial Nursing Association reported in our last issue. Mr. Clifford related his own personal experiences of an illness in the Straits Settlements when there was no nurse available, and the miseries he suffered, only in order to accentuate the contrast when, being again laid aside, he was able to enjoy the services of two of the nurses sent out by the Colonial Nursing Association and received every possible attention. But he went on to mention that his nurses were on duty eight hours at a stretch, and he averred that in a tropical climate the time should not, in ordinary circumstances, exceed six hours. We entirely agree with him, and we do not doubt that his suggestion for four shifts of six hours in place of three shifts of eight hours will receive the consideration it deserves. Now that—as Sir Charles King-Harman pointed out—thanks to the efforts of Mrs. Francis Piggott, the devoted founder of the organisation, the Colonial Nursing Association has become a great and growing power, it is of the utmost importance that the nurses employed under its auspices who have to suffer climatic disadvantages should not run the risk of breaking down from the strain imposed by an unnecessarily prolonged vigil in the sick-room.

### INDISCREET NURSES ON THE WEST AFRICAN COAST.

Although highly satisfactory reports of the excellent work done by individual British nurses in the colonies are sent to us from time to time, there are, unfortunately, exceptions. A few weeks ago the intelligence of the dismissal for misconduct of a nurse in the West Indies who had occupied an important position reached us, and we have now the more unpleasant information, from authoritative sources, that some of the nurses employed on the West Coast of Africa are anything but a credit to their profession, owing to their follies. The situation is, in fact, so serious that the Governments concerned are, we understand, contemplating the discontinuance of the employment of trained nurses from England on the coast. Indeed the Governor of the Gambia has taken positive action, and is employing sisters from a religious order in his territory in consequence of the troubles experienced on the coast, and, if they prove a success, it is more than likely that the exclusion of trained lay nurses from the important centre over which he has control will follow in time. We trust that this will be taken as a note of warning to those whom it most concerns, and that they will awake, before it is too late, to a sense of what is due to their calling and their country.

### THE NEW MATRON OF PORTSMOUTH HOSPITAL.

The appointment of Miss Ethel J. Atkins to the post of matron of the Royal Portsmouth Hospital promises well. Miss Atkins, who was trained at St. Bartholomew's Hospital, has enjoyed a varied and valuable experience. On leaving St. Bartholomew's she went to Birmingham, and was sister for a year at the City Hospital, with 400 beds. She was next appointed assistant matron at the Western Fever Hospital, Fulham, under the Metropolitan Asylums Board, with 450 beds. She relinquished this post in order to undertake the duties of matron at Cuddington Hospital, Banstead, with 60 beds. In July 1898 she was chosen to fill the important office of matron of the Park Fever Hospital at Hither Green, with 550 beds. In 1900 she entered the Army Nursing Service Reserve, and during the war in South Africa was acting superintendent of the Hospital Ships *Lismore Castle* and *Dunera.* In 1901 she returned home and became matron of St. Leonard's Infirmary, Shoreditch, with 500 beds. Here she was head of a staff comprising an assistant matron, 10 sisters, 23 nurses, and 16 probationers. Miss Atkins will have no easy task at Portsmouth, but she will bring to bear upon the difficulties that confront her the wide knowledge of the conditions of nursing which usually develops the broad sense of justice so essential to the maintenance of impartiality.

### FOUR YEARS' TRAINING.

It is proposed that the term of training at Swansea General Hospital should be increased from three to four years. The matron is opposed to the extension of the time, which she believes would render it more difficult for her to obtain probationers. We think that she is probably right. The fourth year of training is as a rule confessedly added in the interests of the hospital rather than in those of the probationers. In large training schools, where the number of candidates is usually far in excess of the vacancies, the longer term may, from every point of view, be sound policy. But in the smaller provincial institutions a term of three years is both more judicious and more fair.

### LADY DUFFERIN'S TRIBUTE TO A NURSE.

Although the Marchioness of Dufferin and Ava was not able to preside at the annual meeting of the Bangor District Nursing Society, County Down, her report as president was read by her daughter, Lady Nicholson. In her comments on the work of the society, Lady Dufferin said that Miss Borlase, the nurse, has "beaten the record," and added, "she has done an amount of work which few people—I will not limit my remark to women—would have either the will or the strength to undertake. An average of 25 visits a day for eleven months represents a continuous steady effort carried on throughout a long period—months in which very little time seems to be set apart for recreation or rest. Moreover, her work is well done, for it has given complete satisfaction to the medical men in attendance on the cases and to the patients themselves." We are glad that Lady Dufferin had the courage to assert the capacity of some women to work as hard as men. It is too frequently assumed that women are invariably prevented in consequence of inferior physical strength from "holding out" as long as the other sex, but even if this may be regarded as the rule, the exceptions are numerous. At the same time we think that one of the speakers rightly urged the need of giving Miss Borlase help. She can hardly be expected to continue to pay 25 visits a day for eleven months, do her work well, and not suffer from the strain.

### A FORWARD MOVEMENT.

It is a considerable time since the Limerick Board of Guardians had under consideration a scheme for improving the system of nursing in the union hospitals. Chiefly because of the expense it was allowed to fall into abeyance, though it was received with general favour. But the Irish Local Government Board are not disposed to permit the order which prohibits the employment of pauper assistants to be set at defiance, and Dr. Biggar, one of the medical inspectors, has lately emphasised the advantages which would follow the establishment of a school for the training of nurses in conjunction with the other hospitals in the city. On this occasion the matter, at Dr. Biggar's instance, was referred to a committee, and we learn that there is now a reasonable prospect of action being taken. At present there is no single institution in Limerick at which a nurse can obtain a certificate to satisfy the requirements of the Local Government Board, and the formation of a training school would doubtless not only enable the guardians to comply with these, but would also be useful to the authorities of Barrington's Hospital, St. John's Hospital, and the County Infirmary.

### NURSES AT ST. ANDREW'S HOME.

There was a pleasant social afternoon at St. Andrew's House Club on Thursday last, and a large number of nurses and friends found their way to the comfortable and artistic quarters in Mortimer Street. Music was given by a ladies' band at intervals, one young lady playing a violin solo, and another a solo on the 'cello. Refreshments were served in the pretty dining-room, gay with Coronation flowers, and most of the visitors took the opportunity after tea of inspecting the building, which has been recently decorated and papered. The green tiles which cover the walls of the kitchens and bathrooms, owing to the fact that they can be washed every week, look delightfully cool and clean. The protections against fire were especially pointed out, owing to the recent fires in high buildings, and it was satisfactory to learn that every two months there is an afternoon devoted to fire drill. Men arrive and test all appliances, water is pumped up and turned on, and every inmate is asked to go down the iron outside staircase so as to be familiar with the exits in case of necessity. The number of members of the club is at present 220, and it is fast increasing.

### A NEW DEPARTURE AT BRISTOL.

The Bristol Board of Guardians applied to the Local Government Board to issue an order providing that the Stapleton and Eastville Workhouse Infirmaries shall be recognised as training schools for nurses qualifying for the post of superintendent nurse under Article III. of the Nursing in Workhouses Act, 1897. To this the Local Government Board answered that they will be prepared, if the guardians so desire, to issue an order similar to one which they issued a few years ago to an infirmary in the north of England, in which it was provided that the infirmary of the workhouse should be deemed to be an infirmary maintaining a resident physician or house surgeon during such period as the medical officer of the workhouse should devote his whole time to the duties of his office. The guardians were of opinion that the communication from the Local Government Board was satisfactory, and resolved to apply for the necessary order.

### NIGHT NURSES ON THE HOSPITAL SHIPS.

Although we allow "A Night Nurse on the Hospital Ships" to state her case in our columns this week, we must call attention to the fact that, while asserting that "in not one detail" was our informant who supplied us with some particulars to which we referred on July 5th "truthful," she does not herself prove the inaccuracy of a single allegation. More to the point were the assertions of two correspondents last week, one of whom said that when she was charge nurse the nurses were allowed a cup of tea in their bedroom, and also to spend the time before retiring to bed at noon on deck, seats with awnings being provided for them. But as "A Night Nurse on the Ships" does not say a word about the cup of tea, and intimates that the nurses, though not forbidden to go on deck, have "to book off punctually," it is possible that other changes may have been made since last year, when "Charge Nurse" was on duty, in addition to the disappearance of the fields in which she used to walk.

### THE NEW HOME AT STOCKTON.

At the monthly meeting of the Stockton and Thornaby Nursing Association last week it was announced that Mr. H. G. Spence had sent a cheque for £100 towards the thousand which is still required by the committee for the purchase and improvements at Barrington House, the new home for the nurses. This handsome contribution had the immediate effect of deciding the committee to issue an appeal to the public to subscribe the remainder of the money, and it was also determined to have an "at home" or other formal opening ceremony at Barrington House next month. A generous gift almost always has a stimulating effect, and is therefore not only valuable in itself, but also in the influence it has upon other people.

### A COMPLIMENT TO A WORKHOUSE MATRON.

At the last meeting of the Ongar Board of Guardians Captain Hervey said that, having visited the workhouse two or three months ago and last year, he was "strongly of opinion that the services of an extra nurse were required in the infirmary." The Board, he continued, "ought not to rely too much on casual persons rendering help which should only be given by trained nurses." Thereupon, a guardian inquired if he meant that there had been any neglect. Captain Hervey, somewhat rashly, replied: "No; and there never will be while you have so excellent a matron." An assurance which seems to have convinced the Guardians that there was no necessity to take any action. But however excellent a matron may be, her excellence is not a sufficient reason for an inadequate nursing staff.

### THE NEW HOME AT BRIDGEWATER.

The permanent memorial of the people of Bridgewater to the late Queen Victoria is the commodious and comfortable Nursing Home, in King Square, which has just been opened by the Hon. Mrs. Stanley. A most pleasing feature in connection with the enterprise is, that at the luncheon which followed the opening ceremony, the honorary secretary was able to announce that the memorial committee were in a position to hand over the Home to the District Training Association free of all encumbrance. To show how the movement had elicited the enthusiasm of the people, the honorary secretary mentioned that a domestic servant in the town had collected from her fellow servants 30s., with which she had purchased a clock and presented it to the Home, while a carpenter made and gave the flower boxes in the windows of the house. Miss Hughes, who was one of the speakers, expressed her gratification that the opening of the Home was to be followed by the engagement of an additional nurse, and as the two nurses have had to attend between them more than 200 cases, there is clearly need for a third. The contributions to the furniture of the Home by private individuals included many valuable gifts.

### QUEEN'S NURSES AND GLOUCESTER GUARDIANS.

The Gloucester Board of Guardians subscribe, with the sanction of the Local Government Board, £150 a year to the District Nursing Society. In these circumstances we do not in the least blame Mr. Wintle, a member of the board, for asking searching questions respecting the services rendered by the Queen's Nurse in return for the substantial grant from the rates. There was no lack of testimony as to the value of the assistance given. The chairman said that the District Nursing Society dealt with many cases that might otherwise have had to go to the workhouse infirmary, and several other members of the board cited instances within their knowledge and in their own parishes, showing that the contribution was well earned. Finally, Mr. Spring observed that in looking through the book of the medical officer he found several entries "nursing attendances," proving that within the last six weeks there had been a great many nurses in attendance in cases of the board's own relief. When Mr. Spring concluded his speech, Mr. Wintle allowed the subject to drop, from which it may be assumed that he was satisfied.

### ISLINGTON INFIRMARY.

For the first time since its opening, Islington Infirmary was *en fête* on Monday last. The occasion was to have been the celebration of his Majesty's Coronation, but that event having unfortunately been postponed, the preparations which had been made were not allowed to be wasted. The Committee had given the medical superintendent *carte blanche* to regale the patients with whatever was reasonable and seasonable, and a very attractive menu was the result, the patients enjoying the good things provided in unmistakable fashion. Dinner being duly disposed of, the afternoon was devoted to a concert of unusual excellence. Owing to the reduction in the number of inmates at this time of the year, a vacant ward was improvised into a concert room, and very well it served its purpose. Great interest was created by the appearance of that old favourite of everyone—Madame Antoinette Sterling. Other ladies who contributed to the enjoyment of their hearers were Mrs. Sydney Brooks and Miss Beatrice May. Mr. Brooks also performed several striking solos, which were much appreciated. The proceedings were unexpectedly interrupted by a short visit in state of the Mayor and Mayoress of Islington. The concert having been brought to a conclusion by the singing of the National Anthem, the visitors repaired to the lawn in front of the medical superintendent's residence, where afternoon tea was served. Quite an army of nurses and probationers were present, headed by Miss Little the matron, and the scene beneath the beautiful old trees, which are such an attraction to the infirmary, became quite gay and picturesque. Later on, when the duties of the day were at an end, a very pleasant evening was passed.

### Lectures to Nurses on Medical Nursing.

By Eveline A. Cargill, M.D.Brux., L.R.C.P.&S.Ed.

### LECTURE II.—DISEASES OF THE DIGESTIVE ORGANS.

### Foods and Feeding.

In cases of severe illness the digestive organs are affected and food has to be administered in quality and quantity to suit the case. Feeding the patient is one of the chief duties of the nurse, and it is often one of her chief difficulties as well. In all cases where a special diet is ordered the nurse should try to get a diet table written out by the doctor, but sometimes she has to be content with vague instructions, and then she must use her own intelligence to arrange a dietary which will suit her patient and carry out the doctor's orders, and to do this she should have an elementary knowledge of dietetics.

In acute diseases, and when there is fever, the digestive powers are weakened, and food to be assimilated must be liquid, bland and non-irritating. For this purpose there is no food like milk. Milk contains all the elements necessary for the repair of the tissues of the body, and these elements are united in a readily digestible form. The quantity required varies with the condition of the patient, from two to four pints daily. Other liquid foods used in illness are meat extracts of various kinds, beef-tea, chicken broth, etc. These are stimulating but not nourishing, and though often useful in addition to milk, they cannot take the place of milk. Beef-tea should not be given when there is looseness of the bowels, as it increases diarrhœa. Liquid food must be given often—every two hours, or at shorter intervals, if only a small quantity can be given at a time; one teaspoonful every quarter of an hour if retained and assimilated is of more value to the patient than large quantities that are not digested, but which load the stomach or may be vomited.

Undiluted milk cannot always be digested. When this is the case it may be diluted with lime-water or soda-water in the proportion of two-thirds milk to one-third soda-water. As a beverage the proportion of soda-water may be increased. Milk sometimes (especially to old people) has to be given warm, and then the exact quantity should be carefully warmed before each time of feeding, as if left standing near the fire for any length of time, it is not so digestible. When there is diarrhœa all food should be given cold.

Starchy foods are often given in illness—arrowroot, cornflour, etc., but are difficult to digest. It is preferable to give some prepared food which is already partially digested, such as Benger's or Mellin's food.

In convalescence from severe illness feeding is often a difficulty, digestion is delicate and no strain must be put upon it, but more food and strengthening food is required. Eggs are suitable, especially raw or lightly boiled. The white of egg is almost pure albumen and therefore very nourishing and is easily digested. Fish is good, especially boiled fish, and later poultry or game, lightly cooked and then pounded or minced, and a little mashed potato. Meat is generally too stimulating for some little time, especially beef, which must be given with great caution after severe illness; in rheumatic or typhoid fever it may cause a relapse, or determine an attack of kidney disease in those predisposed to that trouble. Peptonised foods are useful in delicate digestion, but should not be persisted in too long or they tend to weaken the digestive powers.

A diet consisting mainly of meat is sometimes ordered for obesity, or in some nervous diseases. The amount of meat is generally definitely ordered by the doctor, and must be always served freshly cooked and hot, preferably broiled or first minced and then broiled after pressing together into a cake. During this diet the action of the kidneys requires careful watching, as meat in large quantities throws a great strain on these organs. This is why in kidney disease when it is desirable to limit the work of the kidneys a diet containing little meat is useful. Also in diseases accompanied by emaciation, where it is desired to fatten the patient, a diet with little meat but much fat and starchy foods is ordered. The trouble here is that excess of hydrocarbon food throws a strain on digestion, chiefly because large quantities must be taken. Bread, milk, milk puddings, cream, fat bacon, cod-liver oil, and maltine are given for fattening purposes.

When for any reason, such as obstinate vomiting or gastric ulcer, in cancer of the œsophagus or stomach, and in some hysterical cases, it is impossible or undesirable to give food by the mouth it may have to be given by the bowel as a nutrient enema. Patients vary in their tolerance of enemata, but from two to about fourteen days is as much as one can hope to continue them; the rectum is apt to become irritable at any time. To give a nutrient enema the rectum must be first emptied of fæces, and for this purpose an ordinary soap and water enema should be administered; give one to two pints at body heat (98°), and let it flow in steadily. If it is desired to pass the fluid high up in the rectum, the patient's hips should be raised, and a soft catheter, well oiled, should be inserted and gently pushed up for 6 or 7 inches; no force must be used. Then attach the tube and inject. After the rectum is emptied and has quieted down again inject the nutrient enema as ordered; it generally consists of peptonised beef tea or milk, egg and brandy, in all about 4 ounces. Use a soft catheter with a glass funnel attached; first let all gas escape, then let the nutrient fluid run in very slowly; if injected quickly it will be returned. It requires much patience. Wash the catheter in hot water directly after using and let it lie in cold water till wanted again. Nutrient suppositories are sometimes used; they must be pushed well up the rectum.

Nothing requires more patience, resource and tact than feeding a patient; they will often take medicine when refusing food, and it taxes all a nurse's powers to get prescribed food swallowed, and it is well to have more than one kind of food ready so that if one form is refused another may be taken. The nurse should attend to the patient's fancies as long as there is no real reason against them, but in really important points the nurse must impose her will. Regularity in meal times is most important, especially in convalescence; a patient counts on meals as a diversion and should not be kept waiting. Careful cooking (the nurse should see to it herself if necessary) and dainty serving, with fresh napery, bright silver, etc., and just the right amount of food nicely arranged on the plate helps to stimulate the appetite. Food, as a rule, should be given dry, the liquid taken at the end of the meal and in small quantities, not more than half a pint; other liquid may be taken between meals, but food eaten dry is more easily digested. Food should never be allowed to remain in the room, and the nurse should never take her own meals in the patient's room.

### To Nurses.

**We invite contributions from any of our readers, and shall be glad to pay for "Notes on News from the Nursing World," or for articles describing nursing experiences, or dealing with any nursing question from an original point of view. The minimum payment for contributions is 5s., but we welcome interesting contributions of a column, or a page, in length. It may be added that notices of appointments, entertainments, presentations, and deaths are not paid for, but that we are always glad to receive them. All rejected manuscripts are returned in due course, and all payments for manuscripts used are made as early as possible after** the beginning of each quarter.

### A Visit to the Bergen Leper Hospital.

BY AN ENGLISH NURSE IN NORWAY.

It was my good fortune while staying at the Bergen General Infirmary to become acquainted with the well-known skin specialist and bacteriologist who is in charge of the two leper hospitals now in use in Bergen. Until the year 1895 there were five leper hospitals in Norway: St. Jörgen's Hospital, built about the beginning of the 15th century; Lungegaard's Hospital, opened 1849; Reknes, opened 1861 (both these were done away with in 1895); Reitgjerdet's Hospital, near Throndhjem, opened 1861; the leper hospital in Bergen, opened 1857, and called No. I. in the annual reports. Owing to the introduction of potatoes, and fresh food instead of salt, together with the diminished exposure to wet and cold during the fishing season, leprosy is very much on the decrease now. To my great delight I was invited by the doctor in charge to accompany him on one of his rounds at 9 A.M. The gate was opened by one of the patients and I was shown to the doctor's study, which led into the dispensary. The latter contains a very limited supply of drugs, because of the small variety of medicines required in such a hospital, while the books overflowed their original bounds and were undergoing a methodical re-arranging in a new room by an expert librarian.

### The Wards.

The hospital was started in 1857, and consists of two wings joined together in the middle and at both ends, so as to enclose two courtyards—one wing for the men and the other for the women. Each contains 16 wards, 10 feet high and 18 feet by 18 feet, originally intended to hold seven beds, but now the number of patients is fewer and there are generally three beds in each ward. Here one was reminded of the keynote of the whole system, that these patients, unlike those of a general hospital, are for the most part chronic sufferers and not spending a few weeks in a ward. It is remarkable how well the idea is carried out in consideration of the individuality of each patient. "Messy" treasures were to be seen, though, unlike the dust-covered collections in an English almshouse, where one often cannot find an available square foot to place a cup, they were limited. Groups of faded photographs stuck on a wall by a bed, where the trembling hand could best reach to place them, remind the patients of the dear ones in a distant fjord, and make that corner a sacred home at once. The window-sills contained private plants, although a clever gardener keeps the gardens down to the lake in excellent order. The liberty of an English almshouse is combined with the enforced cleanliness of a hospital.

### Food and Rules.

Large and airy corridors pass along the whole length of the wings, into which the wards all open, eight above and eight below. To ensure safety in case of fire, two large iron doors divide them, so that under necessity they can become three hospitals instead of one. Besides this, at each end of the corridors there is a staircase leading to the ground floor. The kitchen is on the basement, and communicates with the dining-hall on the ground floor by two lifts—one on the men's side and one on the women's. The food is plain and good, far superior to what the patients would have in their own homes, yet, perhaps, poor in the eyes of beef and beer feeding Britons. Rules resembling those in force in English hospitals concerning the bringing of dainties by friends and relations are in force. I very much regret having broken this rule by regarding the place as a union, and taking some biscuits to a leper friend of mine. Of course, I was not aware of my offence at the time, and the courteous toleration on the part of the doctor makes me feel all the more ashamed of myself now. This is not the only instance of liberal government I can quote. The brother of one of the patients told me how, when he went to say good-bye to his sister, before leaving town, he was refused admission on account of being past the visitors' time. It might have been years before he had another chance of seeing her again, but the doctor arrived at the porter's lodge as he was being sent away, and verified the proverb that "the king's face gives grace" by making inquiries and permitting the sister to come to the lodge and take leave there.

### Lepers Encouraged to Enter.

The gate porter is also a shoemaker at a factory, and while he is absent a trustworthy leper holds the key. New cases are not continually dropping in as at a general hospital, and the expense of a porter always on duty would appear to be hardly necessary. It is not compulsory for the patients to enter the hospital, but all means of persuading them to do so are employed by the parish doctors and those in any influential position. To encourage them, the State gave up the first idea of the patients paying a small amount, and allowed them all to come in free of expense. Even then many shrink from leaving their native fjords to go among strangers. Some have suffered from home-sickness so much that the doctors, fearful of consequences, gave permission for them to return to their own homes, but no sooner had some of them done this than they realised that the hospital was not so dreadful after all, and begged to be taken back. Occasionally a holiday of four weeks during the summer is allowed to those who would otherwise get restless.

### Occupations.

In each wing there are two workrooms. The women are encouraged to spend their time in knitting petticoats and other garments. Since this was started they have been much happier and less inclined to quarrel. Needlework is also done, but even those whose hands are knotted can generally do some knitting. The articles made are disposed of by the governor, and half the money obtained is given to the workers. The men make fishing nets, horn combs, and spoons; do carpentering and shoe-making; but though they ought to deliver their work over to the governor, they sometimes manage to sell things on the sly while out on leave in the town, in order to keep the entire profits. Anyone who wishes may help in the garden for the small wages of 30 öre a day. This is just enough to add interest to the work. In the central connecting building there is a schoolroom where a schoolmaster gives lessons to the children; they are also prepared for confirmation by the clergyman. The chapel is above the dining-hall, and gives a tower-like appearance to that part of the building.

### Male and Female Nurses.

The doctor was attended on his rounds by a male and a female nurse. The former was called the cupper, as the duty of cupping the patients fell on him. Some doctors, however, believe that though cupping may ease a patient for the time, it eventually increases the disease in its worst forms. The female nurse carried a charger somewhat resembling a frying-pan, and was held under the limb that was being dressed. The cupper carried a basket containing the necessary lotions and dressings. Any petty quarrels, which occurred more frequently in the women's quarters than the men's, were brought before the doctor, and he judged them or changed room-mates as he considered necessary. No anæsthetics were used—the patient's nerves being to a large extent dead, they felt no pain. It was strange to see them chattering away while pieces of decayed bone were being removed, as if nothing worse were happening to them

### A VISIT TO THE LEPER HOSPITAL, BERGEN.—*Continued.*

than their nails being cut. To say that they never suffer would, however, be a great error. Of the three forms of leprosy the anæsthetic is the most common.

Curable Cases.

One ward in each wing is set apart for cases where there is a hope of obtaining a cure. This is chiefly among children who often arrive with their parents all afflicted with leprosy. One boy, aged 14 years, came with his father and three brothers, all lepers. He had suffered much before admission while herding cattle. After four and a half years' treatment of sea-water baths and cod-liver oil he was discharged as cured. To ensure his being under observation of doctors he was given the post of porter at the General Infirmary, but, finding the restrictions distasteful to him, he went to sea and nothing has been heard of him since. In many of the cured cases steps have been taken to provide work in Bergen so that the patient may be under medical supervision and not exposed to any unfavourable surroundings.

A Difficult Position for a Nurse.

I had removed my outer wraps in order to let my nursing uniform show that I had come to study—not to stare. I am afraid, however, that the precaution was unnecessary and that the patients had lost all natural sensitiveness. The barbaric surroundings under which they had contracted their disease seemed to have had a hardening, if not a demoralising influence, on them. Indifference to personal appearance and loss of self-respect replaces the purifying influence of pain. The very form of their illness tends to harden their senses. I may be mistaken, but this is my impression gathered from conversation with people who have come in daily contact with them. It struck me that a nurse in such a hospital would find it difficult to keep both herself and her patients from callousness. The nurse must cultivate tenderness for her patients, more for what they are than for what they suffer.

St. Jörgen's Hospital.

I was shown into a laboratory where more books were stored away, and a cupboard containing casts of various remarkable cases of leprosy, and then we proceeded to St. Jörgen's Hospital, called after St. George, the patron saint of lepers. It is a most curious old building, of the type of the French hospitals of mediæval times. Like a ship, the central hall has cabins all round. They used to accommodate two patients each—but now only one. It is reported that this old hospital is to be pulled down as soon as three or four patients, who have bought themselves the right of living there for the rest of their lives, are dead. In the meantime a few others are allowed to share their privileges, which consist chiefly of catering for themselves—often starving for six days in order to be able to stuff on the seventh, and indulge in any less foolish idiosyncrasy. It would be better for the town if, instead of pulling down this historic building, the authorities kept it for a show place, and perhaps collected the entrance fees for the much-needed tuberculosis funds. It is a remarkable coincidence that the micro-organisms of the present and passing national scourges should, as I am told, resemble each other in so far that they are the only microbes that dye red in the Gabbet's test. Reknes is now used as a sanatorium for tuberculosis, and Lungegaard's hospital as a home for incurables in connection with the Bergen General Infirmary.

### Outdoor Uniform.

BY ONE WHO WEARS IT.

"Should nurses wear outdoor uniform ?" is a question which, from the nurse's point of view, badly needs answering. To begin with, all district nurses must wear it. It would be an impossibility, going about as they do from house to house, and having to perform the most varied duties for their patients, for them to wear anything but their neat washing frocks and aprons, covered by the large cloak which can be thrown on one side in a moment. Also, outdoor uniform is necessary to all nurses who do not live at the scene of their work, but go backwards and forwards to meals and for sleeping, as it would take up too much time to be continually changing into private clothes. Perhaps, too, there would be no place to keep them. But I think that these two classes are the only nurses who really need to wear it.

A Suggestion to Hospital Nurses.

The hospital nurse may plead that she has no time to change, her two hours off duty pass all too quickly as it is; but in these days of coats and skirts, what is so easy, if the uniform, bodice and skirt are made separately, as to slip off the print skirt and replace it by the tweed, and cover the print bodice by the coat? A sailor hat would complete the neat and inexpensive costume. Certainly in the year 1902 no nurse trained in up-to-date aseptics ought to go out in the same skirt and apron she has been wearing in the wards, to come back with the tail and hem of both, at any rate slightly soiled, and conveying to the wards how many germs the microscope only would reveal. I have seen nurses in rather long print dresses on a wet day, walking along apparently quite unconscious that their skirts were dipping in the mud at every step. Also, it is not fair to the general public that the nurse should convey to the outer world, on the tails of her garments, the germs which must be floating around in a hospital ward, else why our persistent precautions to kill them? The private nurse, also, would find that the coat and skirt, etc., would be quite as inexpensive and take up as little room as the everlasting cloak and bonnet. A great many private patients have to be taken out by their nurses, and I am sure, poor things, they would rather have an unobtrusive person in private garb with them than let all the world know that they were invalids and not able to be out without an escort.

The Question of Appearance.

Some nurses imagine that they look better in outdoor uniform than mufti, and certainly a few do, if their uniform happens to be a neat one and suits them; but a great many nurses, whatever their private opinion on the subject may be, look anything but beautiful in it. A girl, with "endy" untidy, straight hair, which will come out of curl on the slightest trace of damp in the atmosphere, looks simply too terrible in a bonnet on a wet, windy day, lank locks of hair straying about in all directions. If she wore a hat, she could also wear a face veil, which would keep the unruly hair in subjection. Of course, some nurses wear a face veil with uniform, but what looks worse? Some women, who are neat and ladylike in private clothes, look positively appalling in uniform.

Nurses and Nursemaids.

Several pretty nurses have told me that when in the City and West End they invariably get spoken to if in uniform, in spite of the fact that they hurry on their way, looking straight ahead; but when in mufti they can pursue their course peacefully, without any molestations. At one time uniform was a novelty and a distinction, and I remember well about fifteen years ago, long before I was a nurse myself, wishing that I, too, could wear the neat bonnet and

### OUTDOOR UNIFORM.—*Continued.*

cloak flying in the wind, displaying yards of snowy apron and jingling chatelaine. But now, alas! every little nursemaid one meets wheeling the ubiquitous "pram" is decked out with bonnet and white strings in all sorts of fancy artistic and inartistic attempts at uniform.

### The Holidays.

Why do nurses wear uniform in their holidays? No one is obliged to do that. On the score of expense it is urged that it is not worth while getting private clothes for just a fortnight or so out of the year, but really, if one saves up a bit, a single frock and a single hat ought not to be too wildly extravagant. The friends of a nurse will perhaps say, in order to save her feelings, that they do not mind her being in uniform, and perhaps a few do not, but the majority will not care to take her about everywhere labelled. Men, as a rule, hate going out with a girl in uniform, though they may be too polite to tell her so; so I entreat nurses to think of the feelings of their poor brothers before their next holidays.

### The Most Desirable Uniforms.

If matrons come to the conclusion that out-door uniform shall be worn by their nurses, let it at least be one that will not be too noticeable. Everybody turns to look at the fancy light greys and fawns some nurses affect. I think the navy blue uniform of the Queen's nurses is the neatest, most becoming, and least conspicuous I have seen. Finally, if nurses must wear outdoor uniform, and I believe it is a rule still in some hospitals and institutions that it shall be worn, they can try and look neat in it. They can button up their cloaks right to the hem, and see that their bonnet ribbons do not call aloud for renewal, and that their white strings are not too evidently shirking a visit to the wash tub. They can refrain from talking over their cases and their favourite doctors in loud tones to their dearest friend on the tops of buses. They should remember that the man in the street is looking on, all eyes and ears, to pick up any little item which may be a discredit to them.

### For Off-duty Hours.

BY A SISTER.

NURSES in London, at least most of those whom I have known, have two ideas for an afternoon's outing: one, to go and "see the shops," meaning a stroll along Oxford Street, down Regent Street, and back through Bond Street; and the other to visit some hospital, and compare it—much to its disadvantage—with their own *alma mater*. This paper will not be written in vain if it persuades even a few newcomers to London that there are other ways, quite as enjoyable as these, of spending an occasional hour off duty. "Half-days" and "whole-days" are of course taken up, as a rule, by visits to friends, or to a theatre, or some little excursion to places a few miles out, where bicycles can be hired by the adventurous to take them to Hampton Court or Hatfield, or down towards the Surrey Hills or the beautiful Kentish lanes and commons. But just for the two or three hours which come nearly every other day, there are dozens of little expeditions to be made, and sights to be seen, which we should not think of missing if we had come on a visit from Australia. But because we might see them any day we often end by not seeing them at all. It is a constant reproach to us living in London that Americans know more of our city than we do; perhaps this is partly because we unconsciously depreciate the value of what we can get at our very doors for nothing. For all the most wonderful and interesting things in London, even within doors, are open free on some day of the week. And most of them are within a penny 'bus ride of the invaluable "Tube," which makes it so easy nowadays to get from East to West.

### The Old Milestone and Kew.

Yet how many nurses in West London have ever noticed the eighteen-hundred-year-old milestone which the Romans set up along the great north road, and which is now to be seen fixed against the wall of St. Swithin's Church in Cannon Street? And how many East London nurses know the delight of a spring day in Kew Gardens, when the "living green" of the young leaves and grass gives place here and there to wonderful masses of hyacinth, daffodil, or crocus, and the houses are full of the scent of lilies and mignonette?

### The Pictures.

There are so many things to see that one wonders where to begin. Just think how easily a dozen afternoons can be spent in looking at pictures. One's thoughts first turn to the National Gallery; but we have all been there, and have perhaps come away with aching head and tired feet and a dull, confused remembrance of acres of pictures, and without a distinct, clear impression of even one picture Now the great mistake in sight-seeing is to try to do too much; it is much wiser to make up your mind beforehand for what you want to look at and ignore any temptation to wander away from it, and you will return with a clear idea of half a dozen pictures perhaps, and with an interest that will lead you to go again and again. Go to see Raphael's great "Madonna," and compare it with the equally famous ones of Botticelli, Mantegna, and Titian; or spend half an hour over the curious Dutch landscapes of Cuyp and Berghem, or the delightful Spanish boys of Murillo's pictures. Or if you are a lover of Browning, find out what the National Gallery has to show of the work of Fra Lippo Lippi and Andrea del Sarto.

### Portraits.

The National Portrait Gallery, too, which is close at hand, offers a large field of interest. There is sure to be some picture there which particularly appeals to us, though we may not have the pride of seeing one of our own ancestors on the wall. Soldiers, novelists, kings, philanthropists, actresses, there they all are, not hung indiscriminately, but arranged in groups, so that we see all the celebrities of one epoch together, as, for instance, Queen Elizabeth in one room with her soldiers and statesmen, and the most noted women writers of the last century hanging together in another room.

### The Newest Gallery.

The Tait Gallery is worth a visit if it were only for the charming view of the river to be had from its steps. There is the old palace of Lambeth opposite, with its memories of bygone archbishops and kings; the brown-sailed boats moored close under the bridge, and the barges piled high with straw, golden in the afternoon sun, or sweet-smelling hay that brings suddenly to one the remembrance of the far-away home in the country. It all makes up, on a fine day, as beautiful a picture as anything that can be seen inside. Yet turn in, and there is the pick of the pictures of the British painters: Romney's and Reynolds' beauties, Gainsborough's fine ladies, Landseer's dogs and horses, and Turner's landscapes will take more than an afternoon to exhaust. Here the same advice holds good; don't try to see too much, but take into your mind thoroughly two or three pictures, such as Burne-Jones' lovely "Beggar-Maid," or whatever else may fall in with your taste.

### The Wallace Collection.

Suppose you have made up your mind for an hour in

FOR OFF-DUTY HOURS.—*Continued.*

Hertford House to see the Wallace Collection. It is a delightfully easy place to get to, as Manchester Square is only a few minutes' walk from the Bond Street Tube station, and consequently from Oxford Street. You will notice, as you go round, many sightseers, gaping and bored, wandering from room to room, heartily tired of "doing" both the collection and what they are pleased to consider their duty. Try a different plan yourself. Say, for instance, that you are going to see pictures of children, with whom French painters deal so delightfully. There they are on nearly every wall. There is a little picture, a Fragonard, which always fascinates the writer, of a dame school with a dozen tiny urchins in the scantiest of costumes, a demure and severe young mistress threatening the black sheep, or rather lamb, of her flock, who stands before her, finger in mouth, fearing the worst; while another imp, the spoilt favourite, curls round at her side in blissful, sleepy content. When you are tired of the French children, and there are many of them, and very sweet ones too, a little further on is what is surely an ideal of loveliness, Del Sarto's "Holy Family," with a most childlike Child at His mother's knee, looking at two little angels. Close by, and in wonderful contrast, is one of Velasquez' Spanish princes, whom he painted so often, Don Baltasar Carlos, a child about the same age as Del Sarto's, but solemn, Spanish, and stiff with the weight of dignity and embroidery on his small shoulders. Next one comes across a dear English baby on its mother's knee, as Sir Joshua saw it, and near it two pictures so familiar from engravings, and so charming, the "Strawberry Girl" and little "Miss Bowles" with her dewy blue eyes and arms tightly clasping her King Charles. Near them hangs a little Gainsborough lady, in a quaint muslin cap worn under a huge white hat, an older damsel by several years than Sir Joshua's, but not much less lovely. Another of Reynolds' children, of an entirely different character, is a very little St. John, a curious forlorn little figure, sitting on a rock and crying aloud in the wilderness. On another wall are Rembrandt's companion pictures of a rather stolid-looking Dutch boy standing by his father, and of his little sister standing at her mother's knee and wearing a sort of copy of her costume. Very delightful children are in a Holy Family by Rubens; but if one enumerated half the Flemish, Spanish, Italian, and French children in the collection it would be a long list. There is a beautiful French group in marble in one of the smaller rooms that ought not to be missed, which, though it is labelled "Cupid and Psyche," is really a very human and lifelike pair of tiny children kissing each other; and in the same room is a water colour of Eastern boys scampering and hurrying out of school, as boys will in every part of the world.

THE CHINA.

The other treasures of the collection appeal even more than the pictures to some tastes, and certainly deserve a special visit. Even the least covetous can scarcely look at the china without wishing for a decoration of the same sort for her own little den; some tiny blue Sèvres teapot, or pair of plates with dainty landscapes painted on them, or a flower jar of lovely shape. Then there are the enamels, jewellery, metal work, and artistic furniture of the rooms, making together one of the most attractive sights in London.

HISTORIC SPOTS.

But besides the picture galleries, there is a great source of interest in visiting the places connected with famous men or great events, such as Cheyne Walk, with its memories of Carlyle and Rossetti; the Charterhouse, where we half expect to see Colonel Newcome's name on the wall with the names of its other famous scholars; Bankside and Lambeth Palace, especially if we have been reading Besant's fascinating book of South London. Even prosaic Cheapside will recall John Gilpin and Gilbert Becket. The old City churches, many of which are open every day for a couple of hours, are all worth visiting. Everyone knows St. Bartholomew's and St. Saviour's, but not everyone has discovered the Savoy Chapel, the last remnant of an ancient palace; or St. Etheldreda's, just off Holborn, the Norman chapel of the Bishop of Ely, centuries ago, when his Grace's garden, close by, was famous for its strawberries; or the bit of Roman wall in the churchyard of St. Giles', Cripplegate. It is worth while to go there, just to sit in the quiet green place and listen to the chimes playing "Home, Sweet Home," till one fancies oneself a score of miles away from the great city. In short, there is no hour, at least in daylight, when an intelligent dweller in London need say, "I have no where to go, and nothing to see!"

### Everybody's Opinion.

**[Correspondence on all subjects is invited, but we cannot in any way be responsible for the opinions expressed by our correspondents. No communication can be entertained if the name and address of the correspondent are not given as a guarantee of good faith, but not necessarily for publication. All correspondents should write on one side of the paper only.]**

A QUESTION OF FAIR PLAY.

"A LONDON NURSE" writes: I should be glad to know if other nurses, having had a similar experience to mine, have come to the conclusion that they have been used to amuse a good paying patient. In reference to my advertisement for a holiday engagement, I was asked to call on a doctor regarding an appointment as attendant to a gentleman in the neighbourhood of Park Lane, the time being specified. No questions were asked as to my capabilities or training, but I was requested to call again next day, the journey each day costing me 1s. 2d. I was introduced to the patient, who played a part of a game of draughts with me: he asked me a few foolish questions which, of course, being a mental patient, were of no importance. The next candidate, already having arrived, I was requested to say good morning. I have heard nothing further, though by the delay I lost the chance of two other appointments. Of course there is every possibility I was not the favoured candidate, but it was reasonable to suppose I was, since I was asked to call a second time, though I protested at the delay and begged for an answer in writing if the position was already filled.

PRIVATE NURSING HOMES.

"D. AND G." write: Having seen "H.'s" letter in THE HOSPITAL for June 28th, we quite agree that it would be much better if all homes were under Government inspection, as "well-run" homes would then be protected from such undignified abuse as "H.'s" letter implies. We both being on the staff of a nursing home where only fully-trained nurses are taken at salaries varying from £32 to £75 per annum, with a fully-trained lady superintendent at the head, we feel that it is only right to point out that there are some well-administered homes, where cleanliness and diet, as well as nursing, are made special features. As to "money grubbing concerns," can "H." verify his statement by pointing out anyone who has made a fortune out of a nursing home? The medical faculty demands that "homes" should be near to them, consequently the locality they are in causes high rents, rates and taxes, and also tends to make domestic expenses proportionately heavy; this evidently gives rise to the acquisition of "exorbitant fees." We believe that no medical man sends patients to any home unless he has a thorough knowledge of the nursing, diet, and cleanliness of that home. The editor in a note on "The Untrained Lady Superintendent," points out, and very rightly so, that it rests with the public to see that they have the genuine article by only going to homes with fully-trained superintendents at their head. We can but feel sorry for "H." if the experience cited be a personal one.

### NIGHT NURSES ON THE HOSPITAL SHIPS.

"A NIGHT NURSE ON THE SHIPS" writes: A note appears in your paper of July 5th which reads as a complaint from all night nurses on the Hospital Ships. As one of them, may I be allowed to state your information is not correct, and casts most undeserved reproach on our matron, of whose kindness and consideration too much cannot be said. In not one detail has your informant been truthful. In addition to other food provided, the nurses could nor dispose of a quantity of meat such as you mention, and no sympathy is required for the nurses who carry over the supplies. For the patients everything is brought to the ward kitchens (by porters), and there is nothing unusual in a nurse carrying soup, custard or milk to the lockers. The night nurses sleep on shore, where there is fresh air and quiet; very few would care to walk on deck at 7 A.M., but prefer to cross at once and go out after breakfast. As a fact, the matron has never forbidden going on deck, but the nurses must book off punctually—no great hardship. When tired or ill nothing is denied us, if the fact is stated and our wishes made known. Every nurse I have spoken to on the subject is most indignant, and considers it a disgrace her name should be coupled with such absurd accusations. For our matron we all have the most sincere respect, admiration and liking; she does all possible for our comfort and pleasure, and will at once attend to just complaints, however trifling.

### AN APPEAL FROM A PRIVATE NURSE.

"A NURSE" writes: I sincerely hope that "Nurse Marguerite's" appeal for an agitation will meet with no response, or the general public will think that nurses are asking for too much. When a patient pays for two nurses it is unreasonable to ask the friends or relatives to do the work themselves, and she might be thankful to have 12 hours off duty. My plan is, when there are two nurses, to change after the doctor's visit, say at 11, 12, or 1 A.M., the same at P.M. The day nurse can then have her walk, and the night nurse gets a shorter night, and, what is important, a good meal in the middle of the day without upsetting the domestic arrangements. If this is impossible, change and take alternate night and day duty. After many years of nursing in various branches, of which private nursing counts the most, I can tell "Nurse Marguerite" that if that is all she can grumble about she is indeed lucky. We don't go nursing to have our lives made brighter, but to brighten other people's, and I confess I am rather sorry for the poor chronic whose nurse feels it a strain to attend to him or her for 12 hours at a stretch. I should certainly advise "Nurse Marguerite" to give up private nursing if she feels it such a strain, for to be happy in your work you must love it. If this is the case, no nurse calls herself a poor nurse, for it is indeed the happiest calling for a woman. One may be weary and tired often, but where else do you find the warm, life-long gratitude that is met with between nurse and patient? Kind speeches never to be forgotten, from lips that can never speak again, make up for weary hours passed in the sick-room, and carry with them more comfort than can be found in any other walk of life, even if it be so-called enjoyment from January 1st to December 31st.

### Presentations.

COTTAGE HOSPITAL, DOUGLAS, N.B.—Miss Grace Strange, nurse-matron of the Countess of Home's Cottage Hospital at Douglas, N.B., for the last five years, has been the recipient of several handsome presents, including a Queen Anne silver tea service from Lady Home, marble timepiece with ornaments, dressing-case and gold-mounted umbrella from the inhabitants of Douglas, and a gold watch from the miners of Rigside as a token of the esteem with which she is held in the district. Miss Strange has been appointed as first district nurse to Great Harwood. Lancashire.

### TRAVEL NOTES AND QUERIES.

SOMEWHERE ON LIMITED MEANS (Matron).—I fear your terms are prohibitive in England. Write to Hôtel de Bruges, Knokke, Belgium, and offer four francs per day. It is not a very pretty place itself, but is a splendid healthy seaside air, and a grand centre for excursions. Write to THE HOSPITAL for my article on it, published September 1st, 1900, which will give you full particulars. Expense of journey, first-class return, from Tower Bridge to Ostend, 10s. 6d. If you do not like this idea, let me hear again.

### Appointments.

ALTRINCHAM GENERAL HOSPITAL.—Miss Ethel Rees has been appointed night superintendent and Miss A. M. Fulham sister of the men's medical and surgical ward. Miss Rees was trained at the General Hospital, Altrincham, and has since been on the hospital private nursing staff. Miss Fulham was trained at the General Hospital, Altrincham.

BELFAST THRONE HOSPITAL.—Miss A. McGifford has been appointed staff nurse. She was trained at the Ulster Hospital for Women and Children.

BRADWELL SANATORIUM, NEAR CHESTERTON.—Miss Florence Hancock has been appointed matron. She was trained at the City Hospital, Birmingham, and has since been charge nurse at that institution and at the City Infectious Hospital, Worcester.

EAST HAM DIPHTHERIA HOSPITAL.—Miss Bartlett has been appointed charge nurse. She was trained at the Infirmary, Alcester, and was afterwards staff at the Brook Hospital, Shooters Hill. She has also done private nursing at Birkdale.

EASTRY UNION WORKHOUSE HOSPITAL.—Miss Kate C. Atherton has been appointed superintendent nurse. She was trained at the Manchester and Salford Hospital for Skin Diseases, and Crumpsall Infirmary. She has since been doing private nursing, and acting as temporary superintendent nurse at Oulton Infirmary, Lowestoft. Miss Atherton holds the L.O S. certificate.

HASLINGDEN UNION INFIRMARY.—Miss Colbert has been appointed superintendent nurse. She was trained at Chorlton Infirmary, Manchester, and has been in charge of the Llanelly Union Infirmary for upwards of eight years.

HORTON INFIRMARY, BANBURY.—Miss Edith Baldwin has been appointed staff nurse. She was trained at Salop Infirmary, Shrewsbury, and has since done private nursing.

IPSWICH NURSES' HOME.—Miss Kate Hunt has been appointed lady superintendent. She was trained at the Bristol General Hospital where she was afterwards on the district and private nursing staff. She has since been night superintendent and temporary matron at the Walsall and District Hospital, temporary matron at Acton Cottage Hospital, London, temporary assistant matron at the County Hospital, York, and matron of a private surgical hospital Birmingham. Miss Hunt holds the L.O.S. certificate.

KINGSTON AND HESTERCOMBE NURSING ASSOCIATION.—Miss Lavinia Wilson has been appointed district nurse. She was trained at the South Eastern Fever Hospital, London, and holds the Rotunda and L.O.S. certificates for midwifery.

OULTON WORKHOUSE INFIRMARY.—Mrs. Julia Hesse has been appointed superintendent nurse. She was trained at the Woolwich Union Infirmary, where she has since been assistant nurse, and from 1894 head nurse and ward sister. Mrs. Hesse holds the L.O.S. certificate.

PLYMOUTH WORKHOUSE HOSPITAL.—Miss Martha Foster has been appointed night charge nurse. She was trained at Chichester Union Infirmary, and has since been staff nurse at Bolton Union Hospital.

ROYAL PORTSMOUTH HOSPITAL.—Miss Ethel Jessie Atkins has been appointed matron. She was trained at St. Bartholomew's Hospital, London, for three years, and has since been, in succession, sister at the City Hospital, Birmingham; assistant matron at the Western Fever Hospital, Fulham; matron at Cuddington Hospital; matron at Park Hospital, Hither Green; nursing sister in South Africa, and acting superintendent of two hospital ships; and matron at Shoreditch Union Infirmary.

RUGELEY DISTRICT HOSPITAL.—Miss Charlotte Ann Sharp has been appointed matron. She was trained at Rugely District Hospital, and has since been district nurse in the town. She holds the L.O.S. certificate.

SALISBURY INFIRMARY.—Miss Abbey Seal has been appointed sister. She was trained for three years at the Chester General Infirmary, and has since been on the private staff of this institution for twelve months.

UNION HOSPITAL, PONTYPRIDD.—Miss B. Anscombe has been appointed charge nurse. She was trained for three years at Shoreham Infirmary, where she was afterwards charge nurse. She holds the L.O.S. certificate.

### Echoes from the Outside World.

### The King's Convalescence.

The progress of the King has been so satisfactory that on Saturday it was officially announced that the Coronation would take place between the 8th and the 12th of August. At the same time it was intimated that the Coronation procession through the streets of London had been abandoned. On Tuesday His Majesty was able to leave town. He was accompanied by the Queen, and the Royal Princesses were also in the train. On his arrival at Portsmouth he was immediately carried on board the Royal Yacht *Victoria and Albert*. The yacht proceeded to Cowes, off which place she will lie for the present. The King bore the journey without fatigue.

### The Queen's Teas.

The Queen's teas to maid-servants take place almost daily, and will continue to the end of the month. Most of the girls are entertained in small parties because the less numerous gatherings are found to be so much more pleasurable and easier to manage than the larger ones. On Wednesday three hundred general servants had been invited to the Zoological Gardens, and the first part of the time all went well, and the visitors vastly enjoyed rides on the elephants and camels and the inspection of the various houses. But just as the tea was all set out on the lawn the rain came pouring down, and the meal had to be finished under umbrellas, a few of the girls who had no umbrellas retiring under the tables. In the middle of the storm the Bishop of London arrived, cheery and smiling, but terribly wet. The unkindness of the elements, however, did not seem to depress Dr. Ingram any more than the servants, of whom he said "nothing can damp the spirit of London girls," for he stood up, hatless and umbrellaless, and read the Queen's message, as calmly as if the sun had been shining. Friday's girls were more fortunate, Mr. and Miss Alexander receiving 70 at Aubrey House, Kensington, where they had a splendid time, whilst 200 more were at Chelsea Town Hall, where Lady William Lennox's string band played to them and Lady Cadogan distributed the Queen's chocolate. Other teas were given at Bricket Wood, St. Albans, and Dulwich College on Monday, and at Kennington Park, Hampton Court, and Petersham on Tuesday.

### The Queen and the Rani.

The Indian Potentates who are in England just now have been fêted and made much of, but, of course, it has been impossible to accord the same hospitality to any ladies of the suite because of the Eastern prejudice against women appearing in public. The Queen, however, with her accustomed kindness of heart, knowing that the Rani of Pertabgarh, who has accompanied her husband, the Rajah, would naturally like to see the wife of the Emperor of India, sent a special invitation to the Rani to come and visit her at Buckingham Palace. The Rani—who is a purdah lady, and lives always in the greatest seclusion—arrived at the Palace closely veiled, and was received by the Queen quite alone, except that Hon. Charlotte Knollys was present. The Rani was also taken by her husband to Frogmore, by special permission, and laid a tribute to Queen Victoria upon Her Majesty's tomb.

### The Coronation Bazaar.

From a financial point of view the great Coronation Bazaar, held last week at the Botanical Gardens in aid of the Hospital for Sick Children, Great Ormond Street, was a marvellous success, £15,000 being taken the first day, £10 000 the second, and £5,000 the third. This by no means represents the total received, for the Stock Exchange sent a cheque for £1,000, and there were many donations. Queen Alexandra and most of the Royal Family attended on Thursday, notwithstanding the stormy weather, and Her Majesty purchased something from every stall. The whole Bazaar had been beautifully arranged, and the decorations elicited words of admiration from the Queen. An auction on Saturday evening realised a large sum, Dan Leno selling, among other things, a piano and a "princess car." Just before the Bazaar opened, it was decided that raffling should not be allowed.

### The Return of the Generals.

Lord Kitchener's arrival in London on Saturday was made the occasion of a great demonstration, and the streets were lined with an enthusiastic crowd all the way from Paddington to St. James's Palace. The Prince of Wales, in the name of the King, was on the platform to welcome the generals, as well as the Duke of Connaught, the Duke of Cambridge, and Lord Roberts. Amongst the spectators were most of the Indian celebrities. An address was presented from the inhabitants of Paddington, and then the Prince of Wales and the other royalties drove off by themselves, purposely separating themselves from the military procession so that nothing might detract from the ovation given to the victorious heroes. The first carriage contained Lord Kitchener, General French, and General Ian Hamilton, all in their war-worn khaki uniforms, and it was followed by the headquarters staff, with Earl Roberts, and an escort of Life Guards. As the cortège passed Buckingham Palace, Queen Alexandra and a group of the Royal Family were standing bareheaded on the balcony, and the Queen waved her handkerchief to Lord Kitchener as a token of welcome, the other ladies refraining from doing so that there should be no doubt that the Queen joined in the general rejoicings. At St. James's Palace the luncheon to the returning generals was held in the Banqueting Room, and the gold plate was used. After the luncheon Lord Kitchener drove to Buckingham Palace and the King personally bestowed upon him the Order of Merit. Lord Kitchener remained talking some little time with His Majesty. In the evening there were illuminations at several points, notably the Canadian Arch.

### Resignation of Lord Salisbury.

The retirement of Lord Salisbury from the post of Prime Minister was somewhat unexpectedly announced on Monday morning. Rumours had been current for some time that the event was impending, and this gained ground on the appointment of Lord Salisbury's private secretary, Sir Schomberg McDonnell, to the secretaryship of the Office of Works. But it was generally supposed that Lord Salisbury would remain at the head of affairs until after the Coronation. The late Prime Minister is now in his seventy-third year. He sat for fifteen years, first as Lord Robert Cecil and then as Lord Cranborne, in the House of Commons, and has been a member of the House of Lords for thirty-five years. Since the summer of 1885 he has been constantly Prime Minister, save for two brief intervals, and until quite recently he was also Foreign Secretary. The King, who was anxious to mark in some special manner his high sense of the great services which Lord Salisbury has rendered to the State, has conferred on him the Grand Cross of the Royal Victorian Order, the star of which is set in brilliants.

### The New Prime Minister.

After Lord Salisbury had tendered his resignation to the King on Friday, and it had been accepted, His Majesty sent for Mr. Arthur Balfour, who, having in the interval consulted with Mr. Chamberlain and other members of the Salisbury Cabinet, undertook to fill his uncle's place as Prime Minister. Mr. Balfour, who was born on July 25th, 1848, is the eldest son of the late James Maitland Balfour, of Whittinghame, and Lady Blanche Mary Harriet, sister to the late Prime Minister. He entered the House of Commons in 1874, but first came into great prominence as Secretary for Ireland. Under his auspices, and thanks to his courage, tact, and patience, the reign of terror in the sister island was terminated, order restored, distress alleviated, and happier relations between the two countries established. In October 1891, on the death of Mr. W. H. Smith, Mr. Balfour was chosen by the unanimous voice of the Unionist party to succeed him, and since 1895 he has been First Lord of the Treasury and Leader of the House of Commons. The new Prime Minister, who is an ardent golfer, is unmarried.

### A Book and its Story.

### MR. HARLAND'S NEW NOVEL.*

In "The Lady Paramount" Mr. Harland is as charming as ever. When one opens a book by him one expects certain things, and in his new novel these things are, as usual, to be found. Delicate humour, a literary style that is charming, rare subtlety in character drawing, touched with a dainty irony that gives fine piquancy to the delineations. Love and a lady form the well-worn theme of the romance, for with all its pretty fooling it is a veritable romance, genuine and simple enough. Italy holds Mr. Harland, but he loves the grey skies and low tones of his own island too, so here, as in his previous book, "The Cardinal's Snuffbox," he makes the lady of his choice at home in the land of sunshine and flowers, and his hero a stalwart Englishman with Italian blood in his veins in possession of an English country estate. Susanna is not a name that in itself is either romantic or picturesque, but it is the one chosen for the heroine, Countess of Sampaola. Thanks to the author the important detail of a name is forgotten in the vivid personality of its owner, who stands in the first chapter for the reader's inspection in the glare of electric lamps on the sea wall by the Commendatore's private landing-stage in conversation with him. It is late in the evening of a summer's day, and the two are in striking contrast. He, her guardian, tall, thin, dignified; she, his ward, radiant, young, wilful. The morning had dawned on the festa which the Commendatore had ordered in honour of her birthday. "Cannon had been fired; two-and-twenty salvoes, though Susanna had protested that this was false heraldry, and that it advertised her, into the bargain, for an old maid. In the afternoon there had been a regatta. . . . Then in the evening there had been a dinner, followed by a ball, and fireworks in the garden." After the ball had come the interview with her guardian; she had disposed of her duenna "The Baronessa" for the time being, having by a ruse placed her under the Commendatore's escort, until she was safely secured, ponderous, and unsuspecting, on board the launch that would convey her to Susanna's summer palace on Isola Nobile, then, with a quick movement, Susanna had given the order to move to the man in charge, and before the Commendatore could realise the situation, the launch, in spite of fruitless expostulations from the Baronessa, was clear of the bay, and Susanna stood in her evening dress a dazzling vision of loveliness in the artificial moonlight. In the background was the southern garden. "Dim and mysterious in the night stood the outlines of palms and cypresses, of slender eucalyptus trees, oleanders, magnolias, of orange trees, where the oranges hung, and the dark foliage, like dull burning lanterns. Susanna's snowy confection of tulle and satin and silver embroidery was all a-shimmer. She wore a collar of pearls round her throat, and a long rope descended to her waist and was then caught up and fastened at the bosom with an opal clasp. A delicate perfume, like the perfume of violets, came and went in the air near her. She held a great white fluffy fan of white feathers in one hand, and in the other carried loose her long white gloves, and gems sparkled on her fingers." The immediate subject under discussion, one which Susanna, with characteristic *sang froid*, had dared to propound to her guardian, was that of her determination to start at once, this very night, for Venice, "*en route* for England." The Commendatore's faded blue eyes flickered anxiously. "I can't think I am dreaming," he remarked, with a kind of vague plaintiveness; "and, of course, you are not serious. My dear, I don't understand." "Oh, I'm as serious as mathematics," she assured him. She gave her head a little pensive movement of affirmation, and lifted her eyes to his, bright with an expression of trustful candour. "You are a wretch, a wicked irresistible little wretch," he exclaimed. This apparently sudden move of Susanna's was not an unpremeditated one. She had, with the aid of an elderly English lady, Miss Sandus, arranged all the details of her journey long ago when Miss Sandus was Susanna's guest in her palace on the Island of Sampaola. Susanna intended to start for Venice that night on board her yacht, which was in waiting in the bay. With her maid Rosina, in charge of innumerable boxes and bags, and her man, Serafina, who was to act as courier on the journey, and later to resume his white cap and apron as *chef de cuisine* on her arrival in England at the country house that she had taken furnished from the agent of her cousin, Anthony Craford. Once more the Commendatore wrung his hands in desperation and cried, "A young girl, a mere child, a mere—he paused for an adequate definition—a mere irresponsible female orphan! And nobody with power to interfere." Susanna, having rid herself of the Baronessa, whose presence she found fatiguing and her views entirely opposed to her own, which were "intensely modern," while the poor Baronessa's were "irredeemably eighteen-sixty," was now free to start on her "wander year." One more explanation she offered to her guardian and then started off, leaving him alone to bear the brunt of impotent, irritating reflections. "How stupid of me," she said. "Yes, it's essential that my cousin Anthony should never dream who I really am. He must fancy that I am just anybody—till the time comes for me to cast my domino and reveal the fairy princess. So I travel under a *nom de guerre*. I'm a rich widow, charming, dashing, not too disconsolate, and my name—is Madame Fregi." She brought out the last words after a moment's irresolution and marked them by a hazardous little smile." As Fregi was the Commendatore's name he sternly forbade such masquerading. Unabashed Susanna quickly found another one not attached to any aminate creature, being that of a mountain in the island, and therefore to be assumed without permission. As Madame Torrebianca she travelled to England with her new duenna, Miss Sandus, whom she met in town, and took down with her to Craford Manor. The real object of Susanna's escapade was to make the acquaintance of her English cousin, Anthony Craford, with the ultimate intention of restoring to him the Italian property and title that she was holding, and to which he was heir. But through indifference and apathy he had neither the wish nor intention to claim either one or the other. The story was an old, and somewhat involved one and without interest to Anthony himself in spite of a certain perennial impecuniousness which disturbed his friend and agent Adrian Willis far more than the master of Craford himself. Adrian Willis is a grotesquely absurd character, made for comedy, and quite unlike the typical man of affairs of a country gentleman. "A distinctly pleasant-looking, rather fat man, with a smiling, round, pink face, smooth shaven, and noticeable pair of big bright blue eyes." With the arrival of the ladies at the Manor House, with its square and spacious hall, a little hackneyed in type, perhaps, the ceiling and walls panelled in dark well-polished oak, the floor a pavement of broad stone flags, covered for the most part, too, by a faded Turkey carpet, and the narrow windows, small paned and leaded, set in deep stone embrasures, a vast fireplace jutting across the corner, the Craford arms emblazoned in the chimney above, and wide oak staircase leading to the upper storey—the real comedy naturally begins. Till then the action of the play has been centred in one person; now it is a comedy of two, and a very charming one too. We have quoted the description of Susanna, and this of Anthony is of one who makes a very excellent complement to her vivid personality: "He was a tall well set up young man—thirty at a guess—with grey eyes, a wholesome brown skin, and a nose so affirmatively patrician in its high bridge and slender aquilinity that it was a fair matter for remark to discover it on the face of one who actually chanced to be of the patrician order." The fine fastidiousness and pessimistic tendencies of Anthony's temperament find in the presence of the irresistible countess full scope for the gratification of æsthetic dreams and an optimistic vision, to which he had imagined himself dead. In following the fortunes of "The Lady Paramount" and her lover, we hope that our readers, when they secure the book for themselves, will find the same pleasure in its perusal that it has afforded to the reviewer.

* "The Lady Paramount." By Henry Harland. (Publisher: John Lane, London. 1 vol. Price 6s.)

### For Reading to the Sick.

"LET THY PRAYER BE SET FORTH AS THE INCENSE."

PRAY for thy Friends: that every deed of love
May be received and registered above;
Kind words, and patient ways, and soft regards,
All turned in heaven to stores of rich rewards.
Pray for the Sick, who suffer in all lands,
God's prisoners, laid in bonds by His own hands:
That on them all His likeness He would trace,
And grant them special offices of grace.

We read in the vision of the beloved disciple, of a "great multitude which no man can number," and that these all came "out of great tribulation." Then, surely, in every generation, through all time, there must be scattered up and down, "of every nation, and kindred, and tongue," many suffering servants of God. As "heart answereth to heart," to the trial felt by one is oftentimes shared by many, severed and yet united. Let each sufferer then think often of the pains and griefs of others, and give them the truest sympathy; and above all, pray continually for them. We are taught to do this day by day in our Church services. Christ, the true Vine, says of each living branch, "He purgeth it, that it may bring forth more fruit." Nor is it difficult to see how this may be, for the afflicted servant of Christ, drawn apart from the turmoil of the world, ever looking upward to his dear Lord's cross, watching His face of sorrow "marred more than any man's," and bearing on His aching brow the Crown of Thorns,—such an one, while sitting in stillness at Jesus' feet, realises more and more His love in suffering, and discovers thus the secret of bearing patiently his own little cross. And this close communion with Christ in suffering is doing for him what nothing else could have done. Out of that softened heart will spring love, sympathy, patience, hope, yea, all the blessed "fruits of the Spirit," until he in his little measure is like the "Captain of our salvation," being made "perfect through sufferings."—*M.B.B.*

Pray

For widows, orphans, solitaries, wives,
For heartless homes, where Love nor lives nor thrives:
That all the women of this English land
May be a steadfast, noble, saintly band,
Seeking, in all, less to be great than good,
Fashioned after God's type of womanhood.

*C. M. N.*

O Lord Jesus have mercy upon all sufferers. Grant them, continually meditating upon Thy holy life of suffering, to realise in weakness the strength of Thine Incarnation; in pain, the triumph of Thy passion; in poverty, the riches of Thy Godhead; in reproach, the satisfaction of Thy sympathy; in loneliness, the comfort of Thy continual Presence; in difficulty, the efficacy of Thine intercession; in perplexity, the guidance of Thy wisdom; and bring them of Thy mercy, when this suffering life is past, to the glorious kingdom which, by their suffering, Thou didst purchase for all who would take refuge in Thy mediation. Amen.—*R. M. Benson.*

Plead

For the whole race which He has made His own,
For which He intercedes before the Throne.
All useful work, O heart! art thou denied,
While this great Censer waiteth at thy side?
Heap on thine incense, heap it full and free;
He, when He offers it, will think of thee.

### Notes and Queries.

**The Editor is always willing to answer in this column, without any fee, all reasonable questions, as soon as possible.**
**But the following rules must be carefully observed:—**

1. **Every communication must be accompanied by the name and address of the writer.**
2. **The question must always bear upon nursing, directly or indirectly.**

*Apartments.*

(123) If I take a furnished room with cooking and attendance but board myself, am I bound to pay full charges when away for holidays?—*Verity*

Yes, unless you have an agreement to the contrary.

*Imperial Nursing Service.*

(124) Will you kindly give me full particulars as to the qualifications, education, and social status of the members of the new Imperial Military Nursing Service, and tell me to whom candidates should apply?—*M. W.*

See THE HOSPITAL *Nursing Section* for April 5th, 1902, for latest particulars.

*Maternity.*

(125) I am anxious to study maternity, will you kindly tell me the best book to study, and also say if at 32 I am too old to begin?—*M. G. F.*

"A Practical Hand-book of Midwifery," by Francis W. N. Haultain, M.D., is considered one of the best. You are rather older than usual, but there are some lying-in hospitals which will train you.

Will you kindly assist me, through THE HOSPITAL, to obtain instructions as to the rules of Midwives' Registration, and tell me anything which would enlighten me on this question of nursing?—*Anxious.*

The Midwives Bill has not yet been passed by Parliament.

Having just finished a three years' course of training in a general hospital, I am anxious to get maternity training. Will you kindly tell me the cheapest way of doing so, and also say if I could make use in any way of the training I already have?—*M. L. W.*

You might possibly get into the maternity ward of a good Poor Law institution. Advertise or see advertisements.

I am a trained maternity and sick nurse. I was engaged to nurse a lady in her confinement from February 26th. On that day she told me that she had made a mistake as to the date, and I agreed to nurse her from March 26th and to take on other work until that time. A week afterwards she wrote to me saying that she had engaged another nurse, and gave me no reason for doing so. Can I claim any part of my fee, as I refused other cases on her account?—*Dover Nurse.*

Do you know a good solicitor whom you could consult? From the facts stated it seems possible that you could claim not only your full fees but compensation for loss of work and maintenance.

I am a trained monthly nurse and midwife and had arranged to attend a lady at her accouchement. She was confined prematurely five weeks too soon. Some hours later she developed small-pox and died. I gave up three cases and have lost others because the ladies are afraid of small-pox. Also my cloak and dress left in patient's were taken away and spoilt at disinfecting station. Can I claim more than treble fee per week? and can I get compensation for cloak and lost cases?—*R. M.*

You cannot claim more than you say, which we hope you may obtain. Ask the official who removed the cloak, etc., and he will tell you to whom to apply for compensation, and write to the ladies who would have otherwise have employed you, showing the hardship of the case. To claim anything in a court of law would be expensive and would probably not succeed.

*Home.*

(126) Can you tell me where a young girl of 20, just starting pulmonary consumption, could be received for open-air treatment? Her friends could manage to pay from one to two guineas weekly, but could not manage more.—*District Nurse.*

The Maitland House Sanatorium, Kidmore, Reading, takes patients at the terms you offer, or you might inquire at the Mount Vernon Hospital for Consumption, Hampstead.

**Standard Books of Reference.**

"The Nursing Profession: How and Where to Train." 2s. net; post free 2s. 4d.
"Burdett's Official Nursing Directory." 3s. net; post free, 3s. 4d.
"Burdett's Hospitals and Charities." 5s.
"The Nurses' Dictionary of Medical Terms." 2s.
"Burdett's Series of Nursing Text-Books." 1s. each.
"A Handbook for Nurses." (Illustrated). 5s.
"Nursing: Its Theory and Practice." New Edition. 3s. 6d.
"Helps in Sickness and to Health." Fifteenth Thousand. 5s.
"The Physiological Feeding of Infants." 1s.
"The Physiological Nursery Chart." 1s.; post free, 1s. 3d.
"Hospital Expenditure: The Commissariat." 2s. 6d.

All these are published by the SCIENTIFIC PRESS, Ltd., and may be obtained through any bookseller or direct from the publishers, 28 and 29 Southampton Street, London, W.C.

